# Relationship between sleep quality and subjective well-being: resilience as a mediator and belief in a just world as a moderator

**DOI:** 10.3389/fpsyt.2023.1297256

**Published:** 2023-12-07

**Authors:** Peng Su, Mu He

**Affiliations:** ^1^School of Marxism, University of Chinese Academy of Social Sciences, Beijing, China; ^2^College of Marxism, Chongqing Medical and Pharmaceutical College, Chongqing, China; ^3^Institute of Marxism, Central South University, Changsha, China

**Keywords:** sleep quality, subjective well-being, resilience, belief in a just world, college students, common prosperity

## Abstract

**Background/Purpose:**

Sleep quality significantly impacts subjective well-being, yet its underlying mechanisms remain largely unknown from a scholarly perspective. Existing research has inadequately addressed the relationship between sleep quality and the subjective well-being of College students. This study primarily investigates the influence of sleep quality on the subjective well-being of College students and explores the mediating role of resilience and the moderating role of belief in a just world.

**Methods:**

The study sample comprises 3349 enrolled College students. Measures include the Pittsburgh Sleep Quality Index, the Subjective Well-being Scale, resilience scale, and belief in a just world scale. A moderated mediation model is employed to verify the mediating role of resilience and the moderating role of belief in a just world.

**Results:**

(1) Sleep quality among College students is significantly positively correlated with resilience, belief in a just world, and subjective well-being. (2) Sleep quality positively predicts subjective well-being among College students. Resilience among College students serves as a mediator between sleep quality and subjective well-being, while belief in a just world moderates the influence of resilience on subjective well-being.

**Conclusion:**

The results suggest that sleep quality can directly enhance the subjective well-being of College students and can also indirectly affect it through resilience. Additionally, belief in a just world can enhance the promoting effect of resilience on the subjective well-being of College students. These findings may contribute to understanding the impact of sleep quality on the subjective well-being of College students and its pathways. These research findings can serve as a reference for improving the subjective well-being of College students.

## 1 Introduction

Subjective well-being is a vital component of mental prosperity, and continually enhancing the population’s subjective well-being is a pressing concern for nations. Academically, subjective well-being is generally understood as an individual’s subjective evaluation of their overall quality of life based on self-defined life goals or standards, encompassing satisfaction with life, as well as judgments of positive and negative emotions (1). Existing research has established the significant impact of subjective well-being on the lives of college students. Lower subjective well-being in college students is associated with heightened anxiety, depression, and stress (2, 3). And in severe cases, it may lead to self-harming behaviors (4). Conversely, higher subjective well-being in college students is linked to richer social relationships, greater extroversion, friendliness, and a more positive attitude toward choices (5, 6). However, over the past few decades, global adolescents have experienced a decline in subjective well-being and an increase in psychological issues (7). College students are a critical resource for a nation’s future development, making it essential to address the issue of their subjective well-being. The relationship between sleep and the subjective well-being of college students is a widely discussed topic in academia, primarily due to the widespread and challenging issues of sleep insufficiency and irregular sleep patterns among college students (8). Nevertheless, there remains a shortage of research in academia on how sleep affects the subjective well-being of college students, along with a lack of exploration into the underlying mechanisms of how sleep impacts their subjective well-being. This study focuses on sleep quality and its connection to the subjective well-being of Chinese college students, aiming to provide valuable contributions to the research on the subjective well-being of college students.

Sleep quality refers to an individual’s self-satisfaction with various aspects of their sleep experience (9), including seven factors such as subjective sleep quality, duration, and sleep hindrance (10). A decade-long longitudinal study revealed that sleep quality can predict an individual’s subjective well-being, with lower sleep quality leading to reduced subjective well-being (11). According to the restoration theory (12, 13), college students may face challenges or difficulties in their campus life, resulting in low moods and decreased well-being. However, through various coping mechanisms, such as rest, their mindset can ultimately return to a normal state or even a more positive and healthy one. Sleep quality serves as a significant restoration mechanism, helping individuals maintain stable emotions, fostering a more positive and optimistic outlook, and increasing subjective well-being (14, 15). Previous research has found that higher sleep quality is associated with greater positive emotions and stronger subjective well-being in various populations, including adolescents (16), older adults (17), athletes (18), and police officers (19). Nevertheless, there is a scarcity of research specifically analyzing the relationship between sleep quality and the subjective well-being of college students. Hence, this study presents the following hypothesis.

Hypothesis 1: Sleep quality is expected to positively predict the subjective well-being of college students.

Resilience is a positive psychological adaptation, referring to an individual’s ability to maintain or recover their psychological well-being when facing adversity, such as difficulties and stressors (20, 21). Among the variables indirectly influencing subjective well-being, resilience is a significant factor when considering the impact of sleep quality. Existing research has mostly focused on the influence of resilience on sleep quality, while the impact of sleep quality on resilience requires broader investigation (22). Through longitudinal studies, some scholars have found that school interventions aimed at improving students’ sleep quality can significantly enhance their resilience (23). High-quality sleep and longer sleep duration have been shown to significantly boost an individual’s resilience (24). According to emotion regulation theory (25), resilience is an effective emotion regulation mechanism that can reduce negative emotions in individuals, helping them maintain emotional stability or recover to a healthy state, ultimately leading to improved subjective well-being. Moreover, research has also identified a significant positive predictive role of resilience in the subjective well-being of college students (26). College students with higher resilience exhibit greater emotional stability, increased life satisfaction, and stronger subjective well-being (27). In summary, sleep quality can significantly impact resilience, and resilience can significantly influence subjective well-being. However, there is currently limited research in academia on the mediating role of resilience between sleep quality and the subjective well-being of college students. In light of this, this study presents the following hypothesis.

Hypothesis 2: Resilience is expected to mediate the relationship between sleep quality and the subjective well-being of college students.

Belief in a just world is a cognitive belief that individuals hold, indicating their belief that they exist in a just and orderly world where people receive what they deserve (28). According to the Belief in a Just World Theory (29), belief in a just world can be divided into two categories: self-just world belief and general just world belief. Self-just world belief pertains to whether the world is just for oneself, while general just world belief relates to whether the world is just for others and society as a whole. In summary, the stronger an individual’s belief in a just world, the greater their endurance and adaptability in the face of hardship and challenges, leading to fewer negative emotions and increased subjective well-being (30). Research has found that when college students have low belief in a just world, the relationship between financial stress and the risk of suicide among college students worsens (31). Additionally, studies have revealed that college students’ belief in a just world can directly regulate their resilience. The higher their belief in a just world, the stronger their resilience, resulting in better adaptability in adverse situations, such as hardship and challenges, and increased subjective well-being (32). In conclusion, belief in a just world can moderate the resilience of college students, with stronger belief in a just world leading to higher subjective well-being. Nevertheless, current research has not yet yielded results regarding the moderating effect of belief in a just world on resilience and subjective well-being. Hence, this study presents the following hypothesis.

Hypothesis 3: Belief in a just world is expected to moderate the relationship between resilience and the subjective well-being of college students.

## 2 Materials and methods

### 2.1 Participants

This study involved 3,349 college students from Chongqing, China. Among them, 1,560 were male college students (46.6%) and 1,789 were female college students (53.4%). The average age was 19.47 years (SD = 1.42). Inclusion criteria: enrolled college students who provided informed consent to participate in this survey.

### 2.2 Research tools

#### 2.2.1 Pittsburgh Sleep Quality Index (PSQI)

The Pittsburgh Sleep Quality Index developed by Buysse et al. (10) and translated and revised by Liu Xianchen et al. (33) was used in this study. The scale comprises seven factors, including subjective sleep quality, sleep onset latency, sleep duration, sleep efficiency, sleep disturbances, use of sleep medication, and daytime dysfunction. Each factor is scored on a 0–3 scale, and the sum of the scores from the seven factors represents an individual’s sleep quality, with a range of 0–21. A higher score indicates poorer sleep quality. This study employed the commonly used criterion of PSQI ≥ 8, as adopted by Chinese scholars, to determine the presence of sleep disorders. The Cronbach’s α coefficient of this scale in this study is 0.78.

#### 2.2.2 Index of Well-being (IWB)

The Index of Well-being developed by Campbell (34) was used in this study. The scale consists of two factors: overall emotional index and life satisfaction, totaling nine items. Each item is scored on a 1–7 scale, and the sum of the weighted scores from the two factors represents an individual’s overall well-being index, with a range of 2.1–14.7. A higher score indicates stronger subjective well-being. The Cronbach’s α coefficient of this scale in this study is 0.91.

#### 2.2.3 Connor-Davidson Resilience Scale (CD-RISC)

The Connor-Davidson Resilience Scale developed by Connor and Davidson (35) and revised by Campbell-Sills and Stein (36) was used in this study. The revised scale is a single-factor scale comprising ten items. Each item is scored on a 0–4 scale, and the sum of the scores from the ten items represents an individual’s resilience, with a range of 0–40. A higher score indicates stronger resilience. The Cronbach’s α coefficient of this scale in this study is 0.88.

#### 2.2.4 Belief in a Just World Scale (BJW)

In this study, we utilized the Belief in a Just World Scale, which was developed by Dalbert (37). The scale comprises two factors: general belief in a just world and self-belief in a just world, totaling thirteen items. Each item is scored on a 6-point scale, and the average of the scores represents an individual’s belief in a just world score, with a range of 1–6. A higher score indicates stronger belief in a just world. The Cronbach’s α coefficient of this scale in this study is 0.92.

### 2.3 Procedures

Over the course of 1 month, a questionnaire survey was conducted with college students from four universities in Chongqing, China. Four universities were selected as the primary sampling frame, taking into account factors such as university type and administrative department. Cluster sampling was then carried out by distributing questionnaires to 15 colleges through teachers and counselors, resulting in a reasonably representative sample. The survey was conducted anonymously, and all data were kept confidential. After data collection, statistical analysis and moderation mediation effect tests were conducted using SPSS 26.0 and the PROCESS plugin (38). The bias-corrected percentile Bootstrap method was employed, with 5,000 resampled iterations and a 95% confidence interval. This study was reviewed by the Ethics Committee of Chongqing Medical and Pharmaceutical College, in compliance with medical ethical principles and the requirements of the Helsinki Declaration.

## 3 Results

### 3.1 Test for common method bias

This study employed self-report questionnaires, which could potentially lead to common method bias. To address this issue, methods such as reverse scoring and anonymous completion were utilized during the survey. Harman’s single-factor factor analysis was conducted to examine the presence of common method bias. The results of the analysis revealed that six factors had eigenvalues greater than 1. Among them, the variance explained by the first factor was 24.30%, which was below the critical threshold of 40% (39, 40), indicating that severe common method bias was not a significant concern in this study.

### 3.2 Descriptive statistics and correlation analysis

Descriptive statistics and correlation analyses were conducted on college students’ sleep quality, resilience, belief in a just world, and subjective well-being. The analysis results indicate that the detection rate of sleep disorders is 22.22%. As shown in Table 1, PSQI scores are negatively correlated with scores of resilience and subjective well-being (higher PSQI scores indicating poorer sleep quality).

**TABLE 1:** Descriptive statistics and correlation analysis (*r*).

Variable	*$\bar{x}$± s*	1	2	3	4
Sleep quality	5.17 ± 3.60	1			
Resilience	22.02 ± 7.61	−0.330***	1		
Subjective well-being	7.81 ± 2.43	−0.379***	0.456***	1	
Belief in a just world	3.55 ± 0.90	−0.137***	0.303***	0.254***	1

***P < 0.001.

### 3.3 Hypothesis testing

In this study, Model 4 from the PROCESS plugin was utilized to examine the mediating effect of resilience between sleep quality and subjective well-being. The analysis results (see Table 2, M1, M2, M3) showed that sleep quality positively predicted subjective well-being (β = −0.38, *t* = −23.70, *P* < 0.001) and also significantly predicted resilience (β = −0.33, *t* = −20.21, *P* < 0.001). In Model M3, after introducing the mediating variable of resilience, sleep quality continued to positively predict subjective well-being (β = −0.26, *t* = −16.36, *P* < 0.001), and resilience also positively predicted subjective well-being (β = 0.37, *t* = 23.66, *P* < 0.001). Using the bias-corrected percentile bootstrap method, the results of the test indicated a 95% confidence interval of (−0.140, −0.106), excluding 0. This indicated a significant mediating effect of resilience between sleep quality and subjective well-being, accounting for 32.30% of the total effect. Hypotheses 1 and 2 were supported through the analysis.

**TABLE 2:** Moderated mediation model tests.

Predictor Variable	M1:SWB			M2:RS			M3:SWB			M4:SWB		
	**β**	**SE**	**t**	**β**	**SE**	**t**	**β**	**SE**	**t**	**β**	**SE**	**t**
SQ	−0.38	0.02	−23.70***	−0.33	0.02	−20.21***	−0.26	0.02	−16.36***	−0.25	0.02	−16.06***
RS							0.37	0.02	23.66***	0.32	0.02	19.22***
BJW										0.11	0.02	7.12***
RS*BJW										−0.04	0.01	−2.77**
R^2^	0.14			0.11			0.27			0.28		
F	561.27***			408.60***			607.30***			326.09***		

Variables were standardized and substituted into the regression model; SWB, Subjective Well-Being; SQ, Sleep Quality; RS, Resilience; BJW, Belief in a Just World. **P < 0.01, ***P < 0.001.

Furthermore, Model 14 from the PROCESS plugin was employed to test the moderating effect of belief in a just world on the latter half of the mediation process involving resilience. The analysis results (see Table 2, M2, M4) demonstrated that belief in a just world positively predicted subjective well-being, and the interaction term between resilience and belief in a just world significantly predicted subjective well-being. This suggests that belief in a just world can moderate the impact of resilience on subjective well-being. The results of the test indicated a 95% confidence interval of (0.003, 0.021), excluding 0, confirming the significant moderating effect of belief in a just world. Belief in a just world can enhance the impact of resilience on subjective well-being, suggesting that college students with higher belief in a just world experience a stronger positive effect of resilience on subjective well-being compared to those with lower belief in a just world. Hypothesis 3 was supported through the analysis.

**FIGURE 1:**
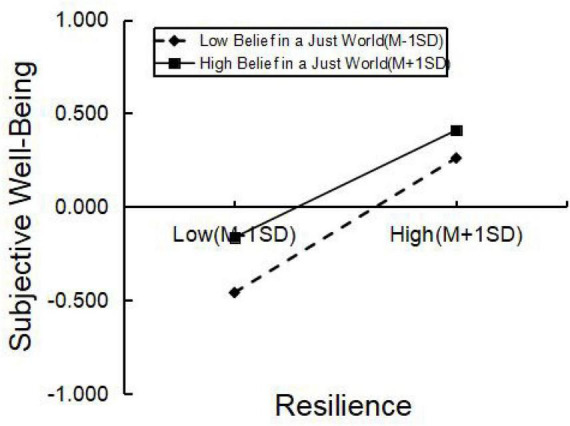
The moderating role of belief in a just world between resilience and subjective well-being.

Additionally, to further explore the moderating effect of belief in a just world, participants were divided into two groups based on belief in a just world scores, specifically one standard deviation above and below the mean. Simple slope analysis was conducted (see Figure 1), revealing that for both high belief in a just world (simple slope = 0.36, *t* = 19.77, *p* < 0.001) and low belief in a just world (simple slope = 0.29, *t* = 11.91, *p* < 0.001), resilience significantly positively predicted subjective well-being. Students with high belief in a just world exhibited higher levels of resilience and subjective well-being than those with low belief in a just world. Furthermore, when belief in a just world was low, the impact of resilience on subjective well-being was even stronger.

## 4 Discussion

This study found that sleep quality can positively predict the subjective well-being of college students. Higher sleep quality is associated with stronger subjective well-being, consistent with previous research in China, the United States, and other countries (16–19). This result can be explained through the lens of the restoration theory. Sleep is a crucial means of recovery and restoration, and high sleep quality effectively dissipates negative emotions in college students, resulting in emotional stability and increased subjective well-being (12). During their university years, individuals often have more flexible schedules, but a lack of guidance and supervision in aspects of healthy living can lead to variable sleep patterns and irregular routines, negatively impacting sleep quality (8, 41). The prevalence of staying up late and nocturnal activities among college students further affects their sleep quality (42). Lower sleep quality fails to alleviate negative emotions in college students, resulting in weaker subjective well-being. Conversely, higher sleep quality helps college students return to a normal state of mind and even attain a more positive and healthy state (14, 15). Therefore, universities should encourage students to prioritize sleep, establish regular routines, and reduce late-night activities to improve sleep quality and subsequently enhance their subjective well-being. In contrast to previous studies, this research uncovers the impact of sleep quality on the subjective well-being of college students and offers a reasonable explanation for the factors contributing to this phenomenon.

Furthermore, this study revealed that resilience plays a mediating role between sleep quality and the subjective well-being of college students. In other words, sleep quality can influence the resilience of college students, subsequently impacting their subjective well-being. This finding addresses the limitations of previous research, as earlier studies often focused solely on the relationship between sleep quality and resilience (24) or the relationship between resilience and subjective well-being (26, 27). The emotion regulation theory provides a strong theoretical framework (43). College students who maintain high sleep quality can adapt well to adversity and stress, indicating better resilience. Resilience, in turn, helps college students maintain positive emotions, higher life satisfaction, and stronger subjective well-being. College students, who have experienced fewer setbacks overall, tend to have lower adaptability and resilience when facing challenges and stressors (44). Poor sleep quality further diminishes college students’ resilience, leading to emotional instability, decreased cognitive functioning, and difficulty overcoming challenges in a healthy psychological state (23). High-quality sleep, on the other hand, strengthens resilience, allowing college students to maintain or restore their mental well-being, ultimately enhancing their subjective well-being (45). Therefore, educational institutions should consider the role of resilience in enhancing the subjective well-being of college students. While promoting high-quality sleep, they should also create opportunities for social practice and encourage students to apply theoretical knowledge to daily life, thereby enhancing their resilience. In contrast to previous research, this study validates the mediating role of resilience between sleep quality and the subjective well-being of college students and provides explanations for these findings.

Additionally, this study found that belief in a just world can moderate the relationship between resilience and the subjective well-being of college students. In simple terms, belief in a just world can enhance the impact of resilience on subjective well-being. This complements previous research, as earlier studies often only analyzed the relationship between belief in a just world and resilience or subjective well-being (30, 32). The belief in a just world theory provides a strong explanation for this result. The stronger an individual’s belief in a just world, the more reasonable explanations they have for various unexpected challenges. They can console themselves more easily when faced with unsolvable problems, leading to greater subjective well-being (46). College students are in the stage of forming their values and generally have higher expectations for social fairness and justice. College students with a higher belief in a just world exhibit greater adaptability and the ability to alleviate negative emotions. They have higher tolerance for the issues in social development, resulting in greater subjective well-being (29). In contrast, when belief in a just world is lower, resilience has a stronger impact on subjective well-being, possibly due to idealism and emotional sensitivity among college students. Their emotions can be influenced by trivial matters, affecting their belief in a just world (47). However, when college students encounter some unfair events or treatment, they may become more accepting of the existence of injustice in reality, thereby increasing their adaptability to adversity and improving their subjective well-being (45). It’s worth noting that when belief in a just world is lower, the impact of resilience on subjective well-being is stronger. However, when belief in a just world is higher, resilience still significantly affects subjective well-being. Furthermore, college students with lower belief in a just world still have a noticeable difference in resilience and subjective well-being compared to those with higher belief in a just world. Therefore, it is essential to make college students understand the essence of fairness and justice, and avoiding exaggeration that might lead to a narrow and one-sided understanding of these concepts. In contrast to previous research, this study confirms the moderating effect of belief in a just world on resilience and the subjective well-being of college students and provides a reasonable explanation for this moderation effect.

Although this study has made some innovative findings, there are still some limitations. Firstly, this research employs cross-sectional data, which has limited explanatory power for causal relationships among variables. Future studies could use longitudinal surveys and experimental methods to further examine causal relationships between variables. Secondly, this study only considers students from universities in a single city and does not sample students from different cities, which could enhance the representativeness of the sample in future research. Thirdly, since previous research has scarcely concentrated on mediation and moderation roles of resilience and belief in a just world within this relationship, this study lacks a scientific reference for sample size calculation. Thus, this study can only rely on similar empirical studies as a basis for determining sample size. Subsequent research can build upon this study to calculate a more scientifically grounded sample size.

## 5 Conclusion

College students are a crucial human resource for promoting national development. However, the academic community has shown minimal attention regarding the link connecting college students’ sleep quality with their subjective well-being, as well as the underlying influencing mechanisms. This study, using a moderated mediation model, reveals that sleep quality positively predicts the subjective well-being of college students. Resilience acts as a mediator between sleep quality and the subjective well-being of college students, while belief in a just world serves as a moderator between resilience and the subjective well-being of college students. These findings contribute to a deeper understanding of the relationship between sleep quality and the subjective well-being of college students, providing valuable insights for enhancing their subjective well-being.

## Data availability statement

The raw data supporting the conclusions of this article will be made available by the authors, without undue reservation.

## Ethics statement

The studies involving humans were approved by the Ethics Review Committee of Chongqing Medical and Pharmaceutical College. The studies were conducted in accordance with the local legislation and institutional requirements. The participants provided their written informed consent to participate in this study.

## Author contributions

PS: Conceptualization, Software, Data curation, Writing – original draft, Writing – review and editing. MH: Investigation, Supervision, Writing – review and editing, Funding acquisition.

## References

[1] Diener E Oishi S Tay L. Advances in subjective well-being research. *Nat Hum Behav.* (2018) 2:253–60. 30936533 10.1038/s41562-018-0307-6

[2] Abdel-Khalek AM Lester D. Constructions of religiosity, subjective well-being, anxiety, and depression in two cultures: Kuwait and USA. *Int J Soc Psychiatry.* (2012) 58:138–45. 21118855 10.1177/0020764010387545

[3] Denovan A Macaskill A. Stress and subjective well-being among first year UK undergraduate students. *J Happ Stud.* (2017) 18:505–25.

[4] Lew B Huen J Yu P Yuan L Wang D-F Ping F Associations between depression, anxiety, stress, hopelessness, subjective well-being, coping styles and suicide in Chinese university students. *PLoS One.* (2019) 14:e0217372. 10.1371/journal.pone.0217372 31260454 PMC6602174

[5] Diener E Seligman MEP. Very happy people. *Psychol Sci.* (2002) 13:81–4. 10.1111/1467-9280.00415 11894851

[6] Bailey KM Frost KM Casagrande K Ingersoll B. The relationship between social experience and subjective well-being in autistic college students: a mixed methods study. *Autism.* (2020) 24:1081–92. 31845592 10.1177/1362361319892457

[7] Marquez J Long E. A global decline in adolescents’ subjective well-being: a comparative study exploring patterns of change in the life satisfaction of 15-year-old students in 46 countries. *Child Indic Res.* (2021) 14:1251–92. 10.1007/s12187-020-09788-8 PMC7611680 34539933

[8] Fischer D McHill AW Sano A Picard RW Barger LK Czeisler CA Irregular sleep and event schedules are associated with poorer self-reported well-being in US college students. *Sleep* (2020) 43:zsz300. 10.1093/sleep/zsz300 31837266 PMC7294408

[9] Nelson KL Davis JE Corbett CF. Sleep quality: an evolutionary concept analysis. *Nurs Forum*. (2022) 57:144–51. 34610163 10.1111/nuf.12659

[10] Buysse DJ Reynolds CF Monk TH Berman SR Kupfer DJ. The Pittsburgh sleep quality index: a new instrument for psychiatric practice and research. *Psychiatry Res.* (1989) 28:193–213. 10.1016/0165-1781(89)90047-4 2748771

[11] Karlson CW Gallagher MW Olson CA Hamilton NA. Insomnia symptoms and well-being: longitudinal follow-up. *Health Psychol.* (2013) 32:311–9. 10.1037/a0028186 22746259

[12] Bonanno GA Romero SA Klein SI. The temporal elements of psychological resilience: an integrative framework for the study of individuals, families, and communities. *Psychol Inquiry.* (2015) 26:139–69. 10.1080/1047840x.2015.992677

[13] Troy AS Willroth EC Shallcross AJ Giuliani NR Gross JJ Mauss IB. Psychological resilience: an affect-regulation framework. *Annu Rev Psychol.* (2023) 74:547–76. 10.1146/annurev-psych-020122-041854 36103999 PMC12009612

[14] Otsuka Y Kaneita Y Itani O Jike M Osaki Y Higuchi S The relationship between subjective happiness and sleep problems in Japanese adolescents. *Sleep Med.* (2020) 69:120–6. 10.1016/j.sleep.2020.01.008 32062038

[15] Weinberg MK Noble JM Hammond TG. Sleep well feel well: an investigation into the protective value of sleep quality on subjective well-being. *Aust J Psychol.* (2015) 68:91–7. 10.1111/ajpy.12098

[16] Short MA Booth SA Omar O Ostlundh L Arora T. The relationship between sleep duration and mood in adolescents: a systematic review and meta-analysis. *Sleep Med Rev.* (2020) 52:101311. 10.1016/j.smrv.2020.101311 32240932

[17] Zhao J Yin H Wang X Zhang G Jia Y Shang B Effect of humour intervention programme on depression, anxiety, subjective well-being, cognitive function and sleep quality in Chinese nursing home residents. *J Adv Nurs.* (2020) 76:2709–18. 10.1111/jan.14472 32749027

[18] Watson A Brickson S. Relationships between sport specialization, sleep, and subjective well-being in female adolescent athletes. *Clin J Sport Med.* (2019) 29:384–90. 10.1097/JSM.0000000000000631 31460952

[19] Baker LD Berghoff CR Kuo JL Quevillon RP. Associations of police officer health behaviors and subjective well-being: the role of psychological flexibility. *Eur J Health Psychol.* (2020) 27:98–108. 10.1027/2512-8442/a000055

[20] Fletcher D Sarkar M. Psychological resilience: a review and critique of definitions, concepts, and theory. *Eur Psychol.* (2013) 18:12–23. 10.1027/1016-9040/a000124

[21] Herrman H Stewart DE Diaz-Granados N Berger EL Jackson B Yuen T. What is resilience? *Can J Psychiatry.* (2011) 56:258–65. 10.1177/070674371105600504 21586191

[22] Arora T Grey I Östlundh L Alamoodi A Omar OM Lam KBH A systematic review and meta-analysis to assess the relationship between sleep duration/quality, mental toughness and resilience amongst healthy individuals. *Sleep Med Rev.* (2022) 62:101593. 10.1016/j.smrv.2022.101593 35462348

[23] Wang J Zhang X Simons SR Sun J Shao D Cao F. Exploring the bi-directional relationship between sleep and resilience in adolescence. *Sleep Med.* (2020) 73:63–9. 10.1016/j.sleep.2020.04.018 32791441

[24] Cooper KB Wilson MR Jones MI. The impact of sleep on mental toughness: evidence from observational and N-of-1 manipulation studies in athletes. *Sport Exerc Perform Psychol.* (2020) 9:308–21. 10.1037/spy0000174

[25] Gross JJ. Emotion regulation: past, present, future. *Cogn Emot.* (1999) 13:551–73. 10.1080/026999399379186

[26] Liu XY Li YH Li WM. A study on the prediction of subjective well-being by psychological resilience among college students. *Chinese J Health Educ.* (2020) 36:234–7. 10.16168/j.cnki.issn.1002-9982.2020.03.009

[27] Yang L Zhao PL Shi ZB. Relationships of parenting style, resilience and subjective well-being in University students. *J Third Military Med Univ.* (2012) 34:2518–21. 10.16016/j.1000-5404.2012.24.001

[28] Lerner MJ Miller DT. Just world research and the attribution process: looking back and ahead. *Psychol Bull.* (1978) 85:1030–51. 10.1037/0033-2909.85.5.1030

[29] Hafer CL Bègue L. Experimental research on just-world theory: problems, developments, and future challenges. *Psychol Bull.* (2005) 131:128–67. 10.1037/0033-2909.131.1.128 15631556

[30] Hafer CL Busseri MA Rubel AN Drolet CE Cherrington JN. A latent factor approach to belief in a just world and its association with well-being. *Soc Justice Res.* (2020) 33:1–17. 10.1007/s11211-019-00342-8

[31] Eunjung Y Eun-Jung S. Does belief in a just world moderate the relationship between financial stress and suicide risk in university students? *Arch Suicide Res.* (2023) 23:660–70. 10.1080/13811118.2022.2039337 35300576

[32] Nartova-Bochaver S Donat M Rüprich C. Subjective well-being from a just-world perspective: a multi-dimensional approach in a student sample. *Front Psychol.* (2019) 10:1739. 10.3389/fpsyg.2019.01739 31417464 PMC6682618

[33] Liu XC Tang MQ Hu L Wang AZ Wu HX Zhao GF Reliability and validity of the Pittsburgh sleep quality Index. *Chin J Psychiatry.* (1996) 29:103–7.

[34] Campbell A. Subjective measures of well-being. *Am Psychol.* (1976) 31:117–24. 10.1037/0003-066x.31.2.117 1267244

[35] Connor KM Davidson JRT. Development of a new resilience scale: the Connor-Davidson resilience scale (CD-RISC). *Depression Anxiety.* (2003) 18:76–82. 10.1002/da.10113 12964174

[36] Campbell-Sills L Stein MB. Psychometric analysis and refinement of the Connor-Davidson resilience scale (CD-RISC): validation of a 10-item measure of resilience. *J Trauma Stress.* (2007) 20:1019–28. 10.1002/jts.20271 18157881

[37] Dalbert C. The world is more just for me than generally: about the personal belief in a just world scale’s validity. *Soc Justice Res.* (1999) 12:79–98. 10.1023/A:1022091609047

[38] Hayes AF Montoya AK Rockwood NJ. The analysis of mechanisms and their contingencies: PROCESS versus structural equation modeling. *Aust Market J.* (2017) 25:76–81.

[39] Podsakoff PM Organ DW. Self-reports in organizational research: problems and prospects. *J Manage.* (1986) 12:531–44. 10.1177/014920638601200408

[40] Hao Z Lirong L. Statistical test and control method of common method bias. *Adv Psychol Sci.* (2004) 06:942–50.

[41] Brown FC Buboltz WC Soper B. Relationship of sleep hygiene awareness, sleep hygiene practices, and sleep quality in university students. *Behav Med.* (2002) 28:33–8. 10.1080/08964280209596396 12244643

[42] Zhu YY Huang JH Tang ZY Liu JY Li X The relationship between bedtime procrastination and daytime sleepiness in college students: a moderated mediation model. *Stud Psychol Behav.* (2022) 20: 797–804.

[43] Yih J Uusberg A Taxer JL Gross JJ. Better together: a unified perspective on appraisal and emotion regulation. *Cogn Emot.* (2019) 33:41–7. 10.1080/02699931.2018.1504749 30058449

[44] Pidgeon AM Rowe NL Stapleton P Magyar HB Lo BC. Examining characteristics of resilience among university students: an international study. *Open J Soc Sci.* (2014) 2:14–22. 10.4236/jss.2014.211003

[45] Yıldırım M Çelik Tanrıverdi F. Social Support, Resilience and Subjective Well-being in College Students. *J Positive Sch Psychol.* (2021) 5:127–35. 10.47602/jpsp.v5i2.229

[46] Bartholomaeus J Strelan P. The adaptive, approach-oriented correlates of belief in a just world for the self: a review of the research. *Pers Individ Diff.* (2019) 151:109485. 10.1016/j.paid.2019.06.028

[47] Bai Q Huang S Hsueh F-H Zhang T. Cyberbullying victimization and suicide ideation: A crumbled belief in a just world. *Comp Hum Behav.* (2021) 120:106679. 10.1016/j.chb.2021.106679

